# Therapeutic Agents for Oxaliplatin-Induced Peripheral Neuropathy; Experimental and Clinical Evidence

**DOI:** 10.3390/ijms22031393

**Published:** 2021-01-30

**Authors:** Takehiro Kawashiri, Keisuke Mine, Daisuke Kobayashi, Mizuki Inoue, Soichiro Ushio, Mayako Uchida, Nobuaki Egashira, Takao Shimazoe

**Affiliations:** 1 Department of Clinical Pharmacy and Pharmaceutical Care, Graduate School of Pharmaceutical Sciences, Kyushu University, Fukuoka 812-8582, Japan; mine.keisuke.035@s.kyushu-u.ac.jp (K.M.); dkobayas@med.kyushu-u.ac.jp (D.K.); inoue.mizuki.049@s.kyushu-u.ac.jp (M.I.); shimazoe@phw.med.kyushu-u.ac.jp (T.S.); 2 Department of Pharmacy, Okayama University Hospital, Okayama 700-8558, Japan; s-ushio@okayama-u.ac.jp; 3 Education and Research Center for Clinical Pharmacy, Osaka University of Pharmaceutical Sciences, Osaka 569-1094, Japan; mayaco@gly.oups.ac.jp; 4 Department of Pharmacy, Kyushu University Hospital, Fukuoka 812-8582, Japan; n-egashi@pharm.med.kyushu-u.ac.jp

**Keywords:** oxaliplatin, peripheral neuropathy, preclinical data, clinical evidence, adverse effects

## Abstract

Oxaliplatin is an essential drug in the chemotherapy of colorectal, gastric, and pancreatic cancers, but it frequently causes peripheral neuropathy as a dose-limiting factor. So far, animal models of oxaliplatin-induced peripheral neuropathy have been established. The mechanisms of development of neuropathy induced by oxaliplatin have been elucidated, and many drugs and agents have been proven to have neuroprotective effects in basic studies. In addition, some of these drugs have been validated in clinical studies for their inhibitory effects on neuropathy. In this review, we summarize the basic and clinical evidence for the therapeutic effects of oxaliplatin. In basic research, there are many reports of neuropathy inhibitors that target oxidative stress, inflammatory response, sodium channel, transient receptor potential (TRP) channel, glutamate nervous system, and monoamine nervous system. Alternatively, very few drugs have clearly demonstrated the efficacy for oxaliplatin-induced peripheral neuropathy in clinical trials. It is important to activate translational research in order to translate basic research into clinical research.

## 1. Introduction

Oxaliplatin is a platinum-based chemotherapeutic agent that is widely used as a standard treatment for colorectal, gastric, and pancreatic cancers, usually combined with other therapeutic agents such as fluorouracil, irinotecan, capecitabine, or tegafur, gimeracil and oteracil, however it often causes severe peripheral neuropathy. Within a few hours to a few days after oxaliplatin administration, acute neuropathy, such as cold sensory disturbance in the limbs and perioral region, appears. In most cases, cold-related acute neuropathy is transient and reversible [1,2]. In addition, sensory deficits as chronic neuropathy, a dose-limiting factor, occur after repeated oxaliplatin administration [2,3]. These neuropathies remain a significant clinical problem with oxaliplatin chemotherapy because they can affect quality of life and lead to drug reductions or discontinuation. Previous reports have suggested that voltage-gated ion channels and transient receptor potential channels are involved in oxaliplatin-induced acute neuropathy [4,5,6]. Chronic neuropathy is thought to be caused by morphological changes in neurons, such as axonal degeneration and damage to neuronal cell bodies [7,8,9]. However, no drugs have been recommended to prevent chemotherapy-induced peripheral neuropathy [10]. Since around 2000, animal models of chemotherapy-induced peripheral neuropathy, including oxaliplatin-induced neuropathy, have been established and reported [11,12,13]. In this study, we reviewed the preclinical and clinical evidence for oxaliplatin-induced peripheral neuropathy.

## 2. Therapeutic Agents in Preclinical Evidence

All articles found in PubMed with the search term “oxaliplatin neuropathy or oxaliplatin neurotoxicity” were surveyed. The last search date was 1 August 2020. Reports that did not include information on therapeutic agents for oxaliplatin-induced peripheral neuropathy and clinical studies were excluded from the analysis. From the surveyed papers, we extracted information on the name and dosage of the drugs that showed statistically significant improvement, their mechanism of action, and the animal species in which they were used.

There were 1657 articles in PubMed for the search term “oxaliplatin neuropathy or oxaliplatin neurotoxicity”. Of these, 127 articles reported on drugs that inhibit oxaliplatin-induced peripheral neuropathy in animal studies. The following is a summary of the drugs had therapeutic effects on oxaliplatin-induced peripheral neuropathy in these basic studies (Table 1).

### 2.1. Antioxidants

Many previous preclinical reports support that oxidative stress plays a role in oxaliplatin-related peripheral neuropathy [27,140,141]. Vitamin C, vitamin E, acetyl L-carnitine, alpha-lipoic acid, and glutathione, which are widely known for their antioxidant effects, have been reported to alleviate the peripheral neuropathy of oxaliplatin in rodents [14,15,16,23,34]. Among the approved drugs, carvedilol, donepezil, dimethyl fumarate, and rosiglitazone have also been reported to reverse the neurotoxicity of oxaliplatin via their antioxidant effects [18,21,22,32]. Moreover, many agents, which have antioxidant effects, inhibit oxaliplatin-caused peripheral neuropathy in preclinical studies [17,19,20,24,25,26,28,29,30,31,33,35,36,37,38].

### 2.2. Anti-Inflammatory Agents

Inflammatory cytokines such as IL-1β, IL-6, and TNF-α were elevated in the dorsal root ganglion (DRG) and spinal cord of oxaliplatin-treated animals, and some agents reduced the peripheral neuropathy symptoms via their anti-inflammatory effects [39,41,42]. Activations of astrocytes and microglia were also observed in the spinal dorsal horn after oxaliplatin administrations, and minocycline, rapamycin, and fluorocitrate inhibited these spinal changes and prevented neurological damage [40,43,44]. 

### 2.3. Sodium Channel Inhibitors

Oxaliplatin-induced acute neuropathy is termed a ‘channelopathy’, as oxaliplatin and oxalate modulated voltage-gated Na^+^ and K^+^ channels in several types of neurons [3,142,143]. For example, oxaliplatin increases the amplitude and duration of compound action potentials interacting with voltage-gated Na^+^ channels in rat sensory neurons [142]. Furthermore, oxaliplatin prolongs the duration of the A-fiber compound action potential related to K^+^ channels [3]. Thus, the effect of oxaliplatin on Na^+^ and K^+^ channels is thought to be involved in acute neuropathy [4]. Many Na^+^ channel inhibitors, such as lidocaine, mexiletine, and lamotrigine have been reported to ameliorate the neuropathic symptoms of oxaliplatin, especially the acute neuropathy [11,45,46,47,48,49].

### 2.4. Potassium Channel Modulators

Glucosinolate glucoraphanin, isothiocyanate sulforaphane, allyl-isothiocyanate, phenyl-isothiocyanate and carboxyphenyl-isothiocyanate inhibited oxaliplatin-induced neuropathy by modulating Kv7 channels [50,51]. It has been reported that tandem pore domains in weak rectifying K^+^ channel (TWIK)-related K^+^ channel 1 (TREK-1) channels are partially involved in the inhibitory effect of riluzole on oxaliplatin-induced peripheral neuropathy [52].

### 2.5. Calcium Channel α2δ Ligands

In animal studies only, gabapentin and pregabalin, which act on *α2δ*, reduced the symptoms of oxaliplatin neuropathy [11,46,48,53,54]. 

### 2.6. Transient Receptor Potential (TRP) Modulators

It has been reported that temperature-sensitive cation channels, such as transient receptor potential ankyrin 1 (TRPA1), transient receptor potential melastatin 8 (TRPM8), and transient receptor potential vanilloid 1 (TRPV1), are involved in oxaliplatin-induced peripheral neuropathy [144,145,146]. It has also been reported that the amelioration of oxaliplatin neuropathy by topiramate, acetazolamide, shakuyakukanzoto, goshajinkigan, eel calcitonin, nifedipine, diltiazem, and mexiletine, is partly due to the downregulation or modulation of TRP channels [55,56,57,58,59,60].

### 2.7. Modulators of Glutamate Nervous System

Some studies indicated that the excessive spinal transmission activities, such as spinal glutamate uptake and spinal N-methyl-D-aspartate receptor subtype NR2B subunit overexpression, are involved in painful neuropathic symptoms related to oxaliplatin [64,69,71]. Riluzole, mirtazapine, ifenprodil, amitriptyline, trifluoperazine, dimiracetam, E2072, and Tat-HA-NR2B9c targeted these glutamatergic nervous systems and showed that oxaliplatin reduced neurotoxicity [64,65,66,67,68,69,70,71]. 

### 2.8. Modulators of Monoamine Nervous System

Monoamines, including noradrenalin and serotonin, play an important role in the descending pain inhibitory system [147]. In also the oxaliplatin peripheral neuropathy animal models, many drugs, such as, duloxetine, fluoxetine, vortioxetine, tandospirone, venlafaxine, xaliproden, clomipramine, and clonidine, also showed analgesic effects by modulating the monoamine nervous system [11,80,81,82,83,84,85,86,87,88,89,90,91,92,93,94]. 

### 2.9. Others

In addition to the above, many other drugs have been identified to reduce oxaliplatin-induced peripheral neuropathy via several therapeutic targets, such as acetylcholine receptors [95,96,97,98], thrombin [106,107], protein kinase C/mitogen-activated protein kinase and extracellular signal-regulated kinase signal [103,104], organic cation transporter [99,100], opioid receptors [46,78,79,80], phosphodiesterase [72,73], hyperpolarization-activated, cyclic nucleotide-gated cation channel [61,62], imidazoline receptors [63], endothelin receptor [74], cannabinoid receptors [75], sigma-1 receptors [76,77], orexin receptors [101], histamine receptors [102], ceramide-sphingosine 1-phosphate [105], chelate of oxalate [11,13], vascular endothelial growth factor [108], and others [48,54,109,110,111,112,113,114,115,116,117,118,119,120,121,122,123,124,125,126,127,128,129,130,131,132,133,134,135,136,137,138,139], at the basic research.

## 3. Therapeutic Agents in Clinical Evidence

We analyzed the articles found in PubMed with the search term “oxaliplatin neuropathy or oxaliplatin neurotoxicity” limited to “clinical trials”. The last search date was 25 June 2020. Reports other than randomized trials and meta-analyses were excluded. Moreover, Information such as the investigational drug and its dosage, chemotherapy received by the patient, study design, number of patients, and results was collected.

There were 533 articles in PubMed for the search term “oxaliplatin neuropathy or oxaliplatin neurotoxicity” limited to “clinical trials”. Of these, 127 articles reported on drugs that inhibit oxaliplatin-induced peripheral neuropathy in animal studies. After excluding reports other than randomized trials and meta-analyses, the authors found 16 reports that they considered to be clinically important. A summarized list of the representative randomized controlled trials and meta-analyses on prophylactic and therapeutic agents for oxaliplatin-induced peripheral neuropathy is shown below in Table 2. 

Duloxetine was tested in a randomized, double-blind, placebo-controlled, cross-over trial, for its ability to treat neuropathy in patients with taxane or platinum [148]. In this study, relative risks (RRs) (95% confidence interval (CI)) of experiencing 30% and 50% pain reduction were 1.96 (1.15–3.35) and 2.43 (1.11–5.30), respectively. A sub-analysis of this study indicates that duloxetine is more effective than taxanes in treating platinum-induced neuropathy.

Intravenous injection of calcium and magnesium is thought to chelate oxalate, and the preventive effects for oxaliplatin-induced peripheral neurotoxicity have been investigated since before [149,150,151,152]. Some studies reported significant inhibitory effects on oxaliplatin-related neuropathy [149,150], some studies did not confirm significant effects [151,152]. The results of a meta-analysis including five studies showed that calcium and magnesium had no significant effect on neuropathy (relative risks (RRs) (95% CI) of incidence of ≥Grade 2 neuropathy and ≥Grade 1 chronic neuropathy were 0.81 (0.60–1.11) and 0.95 (0.69–1.32), respectively.) [153].

Goshajinkigan, a Japanese herbal medicine, has been studied in several clinical trials [154,155,156]. In a randomized controlled trial, goshajinkigan significantly reduced the incidence of Grade 2 or higher neuropathy [154]. In goshajinkigan oxaliplatin neurotoxicity evaluation (GONE) study, the incidence of Grade 2 or higher neuropathy until the 8th cycle was 39 and 51% in goshajinkigan and placebo groups, respectively, which was not statistically significant [155]. This study concluded that goshajinkigan appears to have an acceptable safety margin and a promising effect in delaying the onset of Grade 2 or greater peripheral neuropathy [155]. However, in the interim analysis of goshajinkigan effect for oxaliplatin neurotoxicity inhibition using mFOLFOX6 regimen (GENIUS) study, a multicenter randomized, double-blind, placebo-controlled trial, goshajinkigan significantly increased the incidence of neuropathy [156].

Alpha-lipoic acid and vitamin E, both of which have antioxidant properties, were also examined in clinical trials for their effects on neuropathy in patients using oxaliplatin [157,158,159]. However, neither has been reported to significantly improve neuropathy. Beside, glutathione and calmangafordipir, which also have antioxidant effects, were found to significantly improve neuropathy related oxaliplatin treatment in randomized trials [160,161]. However, the dose of glutathione used in this clinical trial was high (1.5 g/m^2^), and calmangafodipir is undergoing Phase III trials and not approved as a drug at this time. Other drugs such as pregabalin, a general-purpose drug for neuropathic pain, and minocycline, a glial attenuator, have also been tested in clinical trials, but no significant inhibitory effects have been reported [162,163]. 

As described above, few drugs have shown clear therapeutic effects on oxaliplatin-induced peripheral neuropathy in clinical trials. Thus, according to the clinical practice guideline updated by the American Society of Clinical Oncology in 2020, no agents have yet to be recommended for preventing chemotherapy-induced peripheral neuropathy and only duloxetine may be used as a treatment for neuropathy [10].

## 4. Discussion

Recently, the mechanism of oxaliplatin-induced peripheral neuropathy has been elucidated in basic studies, and many drugs and agents targeting this mechanism have been explored and identified for therapy for oxaliplatin-induced peripheral neuropathy. In particular, many inhibitors of neuropathy targeting oxidative stress, inflammatory response, sodium channel, TRP channel, glutamate nervous system, and monoamine nervous system have been identified as candidates for inhibiting oxaliplatin-induced neuropathy in animal research.

Alternatively, very few drugs have shown the efficacy of oxaliplatin for peripheral neuropathy in clinical trials. The American Society of Clinical Oncology’s clinical practice guideline states that only duloxetine can be used for the treatment of chemotherapy-induced peripheral neuropathy [10]. Since duloxetine has been shown to improve pain in clinical trials [148], its use in patients with pain may be beneficial. However, consideration should be given to side effects such as drowsiness, headache, and dizziness. Goshajinkigan and glutathione are drugs that have few side effects, thus they can be considered easy to treat in patients. Goshajinkigan has been reported both to have therapeutic effects on oxaliplatin-induced peripheral neuropathy and not to have the effects [154,155,156]. In an animal study, it has been reported that goshajinkigan does not inhibit the progression of chronic neuropathy, but rather relieves neuropathic symptoms [124]. Therefore, it may be used to relieve symptoms in patients with oxaliplatin-induce neuropathy. 

While many drugs have been reported in basic research as having the potential to inhibit the neuropathy by oxaliplatin, few drugs have developed sufficient evidence in clinical studies. The “valley of death” between basic researches and clinical applications is considered caused by many issues, including the difference between clinical symptoms and animal assessment methods, the cost and time of conducting clinical research, safety considerations in clinical application, and the lack of collaboration between basic and clinical researchers. It is important to promote translational research, that is, to bridge basic research to clinical research.

## Figures and Tables

**Table 1:** The therapeutic agents for oxaliplati-induced peripheral neuropathy in preclinical experiments.

Therapeutic Targets	Therapeutic Agents	Dose	Animals	Symptoms that Showed Improvement	Mechanisms	References
Oxidative stress	Acetyl L-carnitine	60–150 mg/kg	Rats	Mechanical, thermal and cold allodynia	Antioxidant effect	[14]
	Acetyl L-carnitine	50–100 mg/kg	Rats	Mechanical, thermal and cold allodynia	Antioxidant effect	[15]
	Acetyl L-carnitine	100 mg/kg	Rats	Mechanical allodynia	Prevention of deficits in mitochondrial function	[16]
	Alpha-lipoic acid	50–100 mg/kg	Rats	Mechanical, thermal and cold allodynia	Antioxidant effect	[15]
	Calmangafodipir (PledOx^®^)	2.5–10 mg/kg	Mice	Mechanical allodynia and decrease in in IENF density	Antioxidant effect	[17]
	Carvedilol	10 mg/kg	Rats	Mechanical and cold allodynia	Antioxidant and mitoprotective effects	[18]
	Cerium oxide nanoparticles	60 mg/kg	Rats	Decrease in MBP of sciatic nerve and increase in GFAP of spinal cord	Antioxidant effect	[19]
	Cystine and Theanine	280 mg/kg	Rats	Mechanical allodynia and sciatic nervedenegerations	Antioxidant effect (upregulation of glutathione)	[20]
	Dimethyl fumarate	200 mg/kg	Rats	Mechanical allodynia and sciatic nervedenegerations	Antioxidant effect	[21]
	Donepezil	1 mg/kg	Rats	Mechanical allodynia	Recovery of reduction in SOD activity	[22]
	Glutathione	33 mg/kg	Mice	Cold allodynia	Aluminum chelation and antioxidative effect	[23]
	Lycopene	2–4 mg/kg	Rats	Neurodegenerative changes (increases in NCAM and BDNS), and decreases in GFAP and caspase-3) in brain and sciatic nerve	Antioxidant effects (downregulation of SOD, CAT, and GPx), and antiinflamattory effects (downregulation of MAPK14, NF-κB and TNF-α)	[24]
	Melatonin	10 mg/kg	Rats	Locomotor activity, muscular strength, thermal, and mechanical allodynia	Antioxidative effects and inactivations of Bcl-2, caspase 3 apoptotic protein and alterations Cytochrome c release	[25]
	Mn(III) 5,10,15,20-tetrakis(N-n-hexylpyridinium-2-yl)porphyrin (MnTE-2-PyP(5+))	0.3–3 mg/kg	Rats	Mechanical allodynia	Inhibition of nitration and activation of superoxide dismutase in mitochondria, and increase in ATP production in primary nerve sensory axons	[26]
	MnL4 (SOD mimetic compound)	15 mg/kg	Rats	Motor coordination, mechanical and cold allodynia	Antioxidative effects and inactivations of caspase 3/7 in astrocyte	[27]
	Niclosamide	10 mg/kg	Mice	Tactile hypoesthesia and thermal hyperalgesia, IENF density, and demyelination	Antioxidative and antiinflammatory effects	[28]
	Phosphatidylcholine	300 mg/kg	Rats	Mechanical and thermal allodynia	Antioxidative effects (downregulation of malondialdehyde, glutathione, GPx, and SOD in sciatic nerve) and modulation of microglial activities	[29]
	Quercetin	20 mg/kg	Mice	Mechanical allodynia	Antioxidant effect	[30]
	Quercetin	25–100 mg/kg	Mice	Mechanical and cold allodynia	Downregulation of nitric oxide and peroxynitrite	[31]
	Resveratrol	100 mg/kg	Mice	Mechanical allodynia	Antioxidant effect	[30]
	Rosiglitazone	3–10 mg/kg	Rats	Mechanical, cold allodynia and motor coordination	Prevention of catalase impairment	[32]
	Rosmarinic Acid	25–50 mg/kg	Rats	Mechanical and cold allodynia	Reduction of oxidative stress, improvement of mitochondrial function, inhibition of spinal glial cell activation, and suppression of expression of inflammatory markers	[33]
	Rutin	20 mg/kg	Mice	Mechanical allodynia	Antioxidant effect	[30]
	Rutin	25–100 mg/kg	Mice	Mechanical and cold allodynia	Downregulation of nitric oxide and peroxynitrite	[31]
	Silibinin	100 mg/kg	Rats	Mechanical and cold allodynia	Improvement of oxidative alterations	[34]
	SS-20 (mitochondria-targeted peptide)	5–10 mg/kg	Mice	Mechanical allodynia and IENF density	Mitochondrial protection	[35]
	SS-31	5 mg/kg	Mice	Mechanical and cold allodynia	Mitochondria-targeted antioxidant	[36]
	Sulforaphane	5 mg/kg	Mice	Mechanical allodynia and morphological alterations, mitochondrial dysfunction in DRG	Activation of the Nrf2 signaling pathway	[37]
	Vitamin C	50–100 mg/kg	Rats	Mechanical, thermal and cold allodynia	Antioxidant effect	[15]
	Vitis vinifera extract	300 mg/kg	Rats	Mechanical and cold allodynia	Antioxidant effect	[38]
	α-tocopherol	100 mg/kg	Rats	Mechanical and cold allodynia	Improvement of oxidative alterations	[34]
Inflammatory	Bee Venom derived phospholipase A_2_	0.2 mg/kg	Mice	Mechanical and cold allodynia	Suppression of infiltration of macrophages and the increase in IL-1β level in the DRG	[39]
	Fluorocitrate	1 nmol/h (i.t.)	Rats	Mechanical allodynia	Inactivation of microglia	[40]
	Herbal Medicine AC591	10,000–20,000 mg/kg	Rats	Mechanical, cold allodynia, and histological changes in sciatic nerve and DRG	Downregulation of inflammation and immune response	[41]
	Houttuynia cordata Thunb	1000 mg/kg	Rats	Mechanical allodynia	Modulation of Th17/Treg balance by regulating PI3K/Akt/mTOR signaling pathway	[42]
	Minocycline	12.5 nmol/h (i.t.)	Rats	Mechanical allodynia	Inactivation of astrocyte	[40]
	Minocycline	25 mg/kg	Rats	Mechanical allodynia	Inactivation of astrocyte	[43]
	Rapamycin	5 mg/kg	Rats	Mechanical and cold allodynia	Blocking mTOR and decreases in IL-1β, IL-6, and TNF-α	[44]
Na channel	Lidocaine	30 mg/kg	Rats	Cold allodynia	*N/A*	[45]
	Lidocaine	3–10 mg/kg	Rats	Cold allodynia	*N/A*	[11]
	Mexiletine	100 mg/kg	Rats	Cold allodynia	*N/A*	[45]
	Mexiletine	30 mg/kg	Mice	Cold allodynia	*N/A*	[46]
	Lacosamide	10–30 mg/kg	Mice	Mechanical allodynia	*N/A*	[47]
	Lamotrigine	5–10 mg/kg	Mice	Cold allodynia	*N/A*	[48]
	Bromhexine	150 mg/kg	Mice	Tactile, cold allodynia	Inhibition of Nav1.6, Nav1.7, and Nav1.9	[49]
K channel	Glucosinolate glucoraphanin	4.43–119.79 µmol/kg	Mice	Mechanical allodynia	Releasing H_2_S and modulating Kv_7_ channels	[50]
	Isothiocyanate sulforaphane	1.33–13.31 µmol/kg	Mice	Mechanical allodynia	Releasing H_2_S and modulating Kv_7_ channels	[50]
	Allyl-isothiocyanate	1.33–13.31 µmol/kg	Mice	Cold allodynia	Releasing H_2_S and modulating Kv_7_ channels	[51]
	Phenyl- and carboxyphenyl-isothiocyanate	1.33–13.31 µmol/kg	Mice	Cold allodynia	Releasing H_2_S and modulating Kv_7_ channels	[51]
	Riluzole	7.5 mg/kg	Mice	Mechanical and cold allodynia	Involvement of TREK-1 potassium channel	[52]
Ca channel	Gabapentin	10–100 mg/kg	Mice	Mechanical allodynia	Attenuation of cofilin phosphorylation in spinal cord	[53]
	Gabapentin	100 mg/kg	Mice	Cold allodynia	*N/A*	[48]
	Gabapentin	30 mg/kg	Mice	Cold allodynia	*N/A*	[46]
	Gabapentin	300 mg/kg	Rats	Cold allodynia	*N/A*	[11]
	Pregabalin	30 mg/kg	Rats	Mechanical and cold allodynia	*N/A*	[54]
TRP channel	Topiramate	50 mg/kg	Mice	Cold allodynia	Prevention of cytosolic acidification and TRPA1 and TRPV1 modulation in DRG neurons	[55]
	Acetazolamide	50 mg/kg	Mice	Cold allodynia	Prevention of cytosolic acidification and TRPA1 and TRPV1 modulation in DRG neurons	[55]
	Shakuyakukanzoto	100–1000 mg/kg	Mice	Cold allodynia	Inhibition of TRPM8 expression in DRG	[56]
	Goshajinkigan	300–1000 mg/kg	Rats	Cold allodynia	Suppressions of increases in TRPA1 and TRPM8 in DRG	[57]
	Goshajinkigan	1000 mg/kg	Rats	Cold allodynia	Suppressions of increases in TRPA1 and TRPM8 in DRG	[58]
	Eel calcitonin	20 U/kg	Rats	Mechanical and cold allodynia	Inhibition cellular signaling related to TRPA1 and TRPM8	[59]
	Nifedipine	10–30 mg/kg	Rats	Cold allodynia	Downregulation of TRPM8	[60]
	Diltiazem	10–30 mg/kg	Rats	Cold allodynia	Downregulation of TRPM8	[60]
	Mexiletine	10–30 mg/kg	Rats	Cold allodynia	Downregulation of TRPM8	[60]
HCN1/HCN2	MEL57A	1–10 mg/kg	Rats	Mechanical allodynia	HCN1 inhibitor	[61]
	MEL55A	30 mg/kg	Mice	Cold allodynia	Blockade of HCN1/HCN2 Channels	[62]
Imidazoline receptor	2-(1-([1,1’-biphenyl]-2-yl)propan-2-yl)-4,5-dihydro-1H-imidazole) (carbophenyline)	0.1–10 mg/kg	Mice	Mechanical, cold allodynia, and increase in GFAP of spinal cord	I1-imidazoline receptor agonist	[63]
Glutamate	Riluzole	12 mg/kg	Rats	Mechanical allodynia	Suppression of increase in glutamate concentration and decrease in GLT-1 in spinal cord	[64]
	Dimiracetam	100–300 mg/kg	Rats	Mechanical allodynia	Counteraction of NMDA-induced release of glutamate with highest potency in the spinal cord	[65]
	E2072	0.1–1 mg/kg	Mice	Thermal hyperalgesia	Glutamate carboxypeptidase II inhibitor	[66]
	Tat-HA-NR2B9c	50–100 ng (i.t.)	Mice and rats	Mechanical and cold allodynia	NMDA receptor antagonist	[67]
	Mirtazapine	20–30 mg/kg	Rats	Mechanical allodynia	Downregulation of NMDA receptor NR2B subunit	[68]
	Ifenprodil	50 mg/kg	Rats	Mechanical allodynia	NMDA receptor antagonist	[69]
	Amitriptyline	5–10 mg/kg	Rats	Mechanical allodynia	Downregulation of NMDA receptor NR2B subunit	[70]
	Trifluoperazine	0.3 mg/kg	Rats	Mechanical allodynia	Inhibition of CaMKII	[71]
PDE	Tadalafil	10 mg/kg	Mice	Cold, mechanical, and electrical current hypersensitivities, and thermal hypoesthesia.	Increases in blood flow and skin temperature	[72]
	Ibudilast	7.5 mg/kg	Rats	Mechanical allodynia	*N/A*	[73]
Endothelin receptor	Bosentan	100 mg/kg	Mice	Mechanical and thermal hypersensitivity	Antagonism of endothelin ETA and ETB receptors	[74]
Cannabinoid receptor	Cannabidiol	1.25–10 mg/kg	Mice	Mechanical allodynia	*N/A*	[75]
Sigma-1 receptor	E-52862	20–80 mg/kg	Rats	Cold allodynia	Sigma-1 receptor antagonist	[76]
	SA4503	3 mg/kg	Rats	Mechanical allodynia	Sigma-1 receptor agonist	[77]
Opioid receptor	Fentanyl	0.017–0.03 mg/kg	Rats	Mechanical and cold allodynia	*N/A*	[78]
	LOR17 (κ-opioid receptor agonist)	1–20 mg/kg	Rats	Cold allodynia	κ-opioid receptor agonist	[79]
	Morphine	1–3 mg/kg	Rats	Mechanical and cold allodynia	*N/A*	[78]
	Oxycodone	0.3–0.56 mg/kg	Rats	Mechanical and cold allodynia	*N/A*	[78]
	Tramadol	20 mg/kg	Mice	Cold allodynia	*N/A*	[46]
	Tramadol	30 mg/kg	Rats	Cold allodynia	*N/A*	[80]
Monoamines	Amitriptyline	2.5–10 mg/kg	Mice	Cold allodynia	*N/A*	[81]
	Bee venom	0.1 mg/kg	Mice	Mechanical allodynia and IENF density	Activation of the noradrenergic system, via α_2_-adrenegic receptors	[82]
	Bee venom acupuncture	0.25–2.5 mg/kg	Mice	Mechanical and cold allodynia	Activations of spinal opioidergic and 5-HT_3_ receptors	[83]
	Bee venom acupuncture	0.25–1 mg/kg	Rats	Cold allodynia	Activation of the noradrenergic system	[84]
	Bee Venom derived phospholipase A_2_	0.2 mg/kg	Mice	Mechanical and cold allodynia	Activation of the noradrenergic system, via α_2_-adrenegic receptors	[85]
	Clomipramine	2.5 mg/kg	Rats	Cold allodynia	*N/A*	[11]
	Clonidine	0.1 mg/kg	Mice	Mechanical allodynia and spinal p-p38 MAPK expression	α_2_ adrenoceptor agonist	[86]
	Duloxetine	30–60 mg/kg	Mice	Mechanical and cold allodynia	Activating spinal α_1_-adrenergic receptor	[87]
	Duloxetine	30 mg/kg	Rats	Cold allodynia	*N/A*	[80]
	Duloxetine	2.5 mg/kg	Mice	Cold allodynia	*N/A*	[88]
	Fluoxetine	20 mg/kg	Rats	Mechanical and cold allodynia	Blockade serotonergic 5-HT_2C_ receptor	[89]
	Melittin (major content of bee venom)	0.5 mg/kg	Mice	Mechanical and cold allodynia	Activating the spinal α_1_- and α_2_-adrenergic receptors.	[90]
	Morphine	2–5 mg/kg	Mice	Mechanical and cold allodynia	Activations of spinal opioidergic and 5-HT_4_ receptors	[83]
	NLX-112	0.1–5 mg/kg	Mice	Mechanical allodynia	5-HT_1A_ receptor agonist	[91]
	Pregabalin	30 mg/kg	Rats	Cold allodynia	*N/A*	[80]
	Scolopendra subspinipes	0.5%/20 µL (acupoint treatment)	Mice	Mechanical allodynia	Activation of spinal α_2_-adrenoceptor	[92]
	Tandospirone	1–3 mg/kg	Mice	Mechanical allodynia and mast cell migration	5-HT_1A_ receptor agonist	[93]
	Venlafaxine	7.5 mg/kg	Rats	Cold allodynia	*N/A*	[11]
	Vortioxetine	1–10 mg/kg	Mice	Mechanical and cold allodynia	Increases in NA and 5HT in brain	[94]
	Xaliproden	0.3–3 mg/kg	Mice	Mechanical allodynia and mast cell migration	5-HT_1A_ receptor agonist	[93]
Acetylcholine receptor	Citicoline (cytidine-5’-diphosphate- choline; CDP-choline)	1–2 µmol (i.c.v.)	Rats	Mechanical allodynia	Involvement of α7 nAChRs, and interaction between GABAergic and cholinergic system	[95]
	(R)-ICH3	30 mg/kg	Rats	Mechanical and cold allodynia	α7 nAChR agonist	[96]
	PNU-282987	30 mg/kg	Rats	Mechanical and cold allodynia	α7 nAChR agonist	[96]
	αO-Conotoxin GeXIVA 1,2	32–128 mg/kg	Rats	Mechanical and cold allodynia	Antagonism of the α9α10 nAChR	[97]
	α-conotoxin RgIA	2–10 nmol (i.m.)	Rats	Mechanical, cold allodynia, and morphological changes of DRG	α9α10 nAChR antagonist	[98]
OCT2	Dasatinib	15 mg/kg	Mice	Mechanical allodynia	Inhibition of platinum accumulation via OCT2	[99]
OCTN1	Ergothioneine	15 mg/kg	Rats	Mechanical allodynia	Inhibition of OCTN1 and decrease in platinum accumulation in DRG neurons.	[100]
Orexin receptor	Orexin-A	0.1–1 nmol (i.c.v.)	Mice	Mechanical allodynia	Orexin type-1 receptor agonist	[101]
Histamine receptor	S 38093	0.3–3 mg/kg	Rats	Cold allodynia	Histamine H3 receptor agonist	[102]
PKC/MEK/ERK	Trametinib	0.5 mg/kg	Mice	Mechanical and cold allodynia	Inhibition of the MEK/ERK pathway	[103]
	Tamoxifen	10–30 mg/kg	Mice	Mechanical and cold allodynia	Inhibition of PKC/ERK/c-Fos pathway in spinal cord	[104]
	PD0325901	10–30 mg/kg	Mice	Mechanical and cold allodynia	Inhibition of MEK1/2	[104]
Ceramide-sphingosine 1-phosphate	FTY720	0.01 mg/kg	Rats	Mechanical allodynia	Modulation of ceramide-S1P R1	[105]
Oxalate	Calcium gluconate	0.5 mmol/kg	Mice	Cold allodynia	*N/A*	[46]
	Calcium	0.5 mmol/kg	Rats	Cold allodynia	*N/A*	[13]
	Magnesium	90 mg/kg	Rats	Cold allodynia	*N/A*	[11]
	Magnesium	0.5 mmol/kg	Rats	Cold allodynia	*N/A*	[13]
Thrombin activity	Thrombomodulin alfa	0.1–1 mg/kg	Rats	Mechanical allodynia	Activation of TAFI and protein C by modulating thrombin activity	[106]
	Warfarin	1 mg/kg	Mice and rats	Mechanical allodynia	Upregulation of HMGB1	[107]
	Dabigatran	75 mg/kg	Mice and rats	Mechanical allodynia	Upregulation of HMGB1	[107]
	Rivaroxaban	10 mg/kg	Mice and rats	Mechanical allodynia	Upregulation of HMGB1	[107]
VEGF	Bevacizumab	1–15 mg/kg	Rats	Mechanical allodynia	Anti VEGF-A effect	[108]
Others	17α-hydroxyprogesterone caproate	10 mg/kg	Rats	Mechanical and cold allodynia	Reduction of ATF-3, c-Fos, GFAP, Iba-1, IL-1β and TNFα in DRG and spinal cord	[109]
	Allopregnanolone	4 mg/kg	Rats	Motor dysfunction and electrophysiological assesment of motor nerves	*N/A*	[110]
	Alogliptin	10 mg/kg	Rats	Mechanical allodynia and sciatic nervedenegerations	Neuroprotective effects	[111]
	Aqueous Extract of Forsythia viridissima	100 mg/kg	Mice	Mechanical allodynia and decrease in IENF density	*N/A*	[112]
	Aqueous extract of Forsythiae suspensa fruits	50–100 mg/kg	Mice	Mechanical allodynia and decrease in IENF density	*N/A*	[113]
	Aqueous extract of Lithospermi Radix	250 mg/kg	Mice	Mechanical allodynia	Attenuation of spinal microglia and astrocyte	[114]
	Aripiprazole	10 mg/kg	Mice	Mechanical allodynia	*N/A*	[115]
	Astragali radix	100–300 mg/kg	Rats	Mechanical and thermal allodynia	Reductions of morphometric and molecular alterations in peripheral nerve and DRG, and inactivation of microglia and astrocytes in spinal cord and brain	[116]
	Benztropine	10 mg/kg	Mice	Mecahnical, cold allodynia, and demyelination in sciatic nerve	*N/A*	[117]
	Ceftriaxone	200 mg/kg	Mice	Mechanical allodynia	*N/A*	[115]
	Cinnamomi Cortex	100–400 mg/kg	Rats	Cold allodynia	Attenuation of spinal microglia and astrocyte, and downregulation of IL-1β and TNF-α	[118]
	Cryptotanshinone	10–30 mg/kg	Mice	Cold allodynia	*N/A*	[119]
	Curcumin	10 mg/kg	Rats	Neurodegeneration in sciatic nerve	Downregulation of neurotensin and platinum concentrations in sciatic nerve	[120]
	Elcatonin	20 U/kg	Rats	Mechanical and cold allodynia	*N/A*	[54]
	Exenatide	0.1 mg/kg	Rats	Mecahnical, cold allodynia, and demyelination in sciatic nerve	Neuroprotective effects	[121]
	Fulvestrant	5–10 mg/kg	Rats	Mechanical allodynia and sciatic nervedenegerations	Neuroprotective effects	[122]
	Goshajinkigan	300–1000 mg/kg	Mice	Mechanical and cold allodynia	*N/A*	[123]
	Goshajinkigan	300–1000 mg/kg	Rats	Mechanical and cold allodynia	*N/A*	[124]
	Hirudin	10 mg/kg	Mice	Mechanical allodynia	Downregulation of p38, HIF-1α and MMP-9/2	[125]
	HM01	10–30 mg/kg	Rats	Nerveconductionvelocity of digital nerve, caudal nerve and IENF density	Ghrelin agonist	[126]
	Melatonin	3–10 mg/kg	Mice	Mechanical and cold allodynia	Antioxidant effect, improvement of mitochondrial function, activation of autophagy pathway, and anti-apoptotic effect	[127]
	Metformin	250 mg/kg	Rats	Mecahnical, cold allodynia, decrease in IENF density, and increase in GFAP of spinal cord	*N/A*	[128]
	Metformin	250 mg/kg	Mice	Mechanical allodynia	Decreases in ATF-3 and c-Fos expressions in spinal cord and DRG	[129]
	Neurotropin (a non-protein extract derived from the inflamed skin of rabbits inoculated with vaccinia virus)	100–200 U/kg	Rats	Mechanical and cold allodynia	Monoaminergic descending pain inhibitory system via Gi protein-coupled receptors	[130]
	Neurotropin (a non-protein extract derived from the inflamed skin of rabbits inoculated with vaccinia virus)	200 U/kg	Rats	Mechanical allodynia	Neuroprotective effects	[131]
	Ninjin’yoeito	1000 mg/kg	Mice	Mechanical and cold allodynia	*N/A*	[132]
	Palmitoylethanolamine	30 mg/kg	Rats	Mechanical and cold allodynia	Neuroprotective effects and glia-activation prevention	[133]
	Phenytoin	5–10 mg/kg	Mice	Cold allodynia	*N/A*	[48]
	Processed aconite root	1000 mg/kg	Mice	Mechanical and cold allodynia	*N/A*	[134]
	Retigabine	5–10 mg/kg	Mice	Cold allodynia	*N/A*	[48]
	Salmon calcitonin	20 U/kg	Rats	Mechanical and cold allodynia	*N/A*	[135]
	Salvia miltiorrhiza root extract (Danshen)	300–600 mg/kg	Mice	Cold allodynia	*N/A*	[119]
	Tanshinone IIA	25 mg/kg	Rats	Mecahnical, cold allodynia, and demyelination in sciatic nerve	Mitochondrial protection and autophagy promotion	[136]
	Tanshinone IIA	10 mg/kg	Mice	Cold allodynia	*N/A*	[119]
	Topiramate	100 mg/kg	Rats	Mechanical allodynia, dischange in nerve sensory conduction velocity, caudal nerve fibers density, and IENF density	*N/A*	[137]
	Water extract of Lepidium meyenii root	10,000 mg/kg	Rats	Mechanical allodynia	*N/A*	[138]
	Wen-luo-tong	Paws and tails were soaked in 0.6 g/mL solution for 20 min	Rats	Mechanical allodynia	Reductions of histological dischange in DRG and glial activation in the spinal dorsal horn	[139]

Abbreviations: 5-HT, serotonin; Akt, protein kinase B; ATF-3, activating transcription factor 3; ATP, adenosine triphosphate; CAT, catalase; CaMKII, calmodulin-dependent protein kinase II; DRG, dorsal root ganglia; ERK, extracellular signal-regulated kinase; ETA, endothelin A; ETB, endothelin B; GFAP, glial fibrillary acidic protein; GLT-1, glutamate transporter 1; GPx, glutathione peroxidase; HCN1, hyperpolarization-activated, cyclic nucleotide-gated cation channel 1; HCN2, hyperpolarization-activated, cyclic nucleotide-gated cation channel 2; HIF-1, hypoxia inducible factor 1; HMGB1, high mobility group box 1; Iba-1, ionized calcium binding adaptor protein 1; i.c.v., intracerebroventriculary; IENF, intra-epidermal nerve fibers; IL-1β, interleukin-1 beta; IL-6, interleukin-6; i.m., intramuscular; i.t., intrathecal; MAPK14, mitogen-activated protein kinase-14; MBP, myelin basic protein; MEK1/2, mitogen-activated protein kinase kinases 1 and 2; MMP9/2, matrix metalloproteinase-9 and -2; mTOR, mammalian target of rapamycin; nAChR, nicotinic acetylcholine receptor; NF-κB, nuclear factor kappa-B; NMDA, N-methyl-D-aspartate; OCT2, organic cation transporter 2; OCTN1, organic cation transporter novel type 1; PDE, phosphodiesterase; PI3K, phosphatidylinositol-3 kinase; PKC, protein kinase C; SOD, superoxide dismutase; S1P, sphingosine-1-phosphate; TAFI, thrombin-activatable fibrinolysis inhibitor; TNF-α, tumor necrosis factor-α; TREK-1, tandem pore domains in weak rectifying K^+^ channel (TWIK)-related K^+^ channel 1; TRPA1, transient receptor potential ankyrin 1; TRPM8, transient receptor potential melastatin 8; TRPV1, transient receptor potential vanilloid 1; VEGF, vascular endothelial growth factor.

**Table 2:** The therapeutic drugs for oxaliplati-induced peripheral neuropathy in clinical experiments.

Investigational Drug	Dose	Chemotherapy	Study Design	Patient Number	Summary	References
Duloxetine	60 mg/day (30 mg/day for the first week)	Taxane or platinum	Randomized, double-blind, placebo-controlled, cross-over	231	RRs (95% CI) of experiencing 30% and 50% pain reduction were 1.96 (1.15–3.35) and 2.43 (1.11–5.30), respectively.	[148]
Calcium and magnesium	Calcium gluconate, 1 g; magnesium sulfate, 1 g (pre- and post-oxaliplatin)	Oxaliplatin	Randomized, double-blind, placebo-controlled	102	Significant improvements in incidence of ≥ Grade 2 neuropathy, oxaliplatin-specific scale, and acute muscle spasms	[149]
	Calcium gluconate, 1 g; magnesium sulfate, 1 g (pre- and post-oxaliplatin)	Oxaliplatin	Randomized, double-blind, placebo-controlled	139	No significant differences in time to treatment discontinuation	[150]
	Calcium gluconate, 1 g; magnesium sulfate, 1 g (pre- and post-oxaliplatin, or pre-oxaliplatin)	Oxaliplatin	Randomized, double-blind, placebo-controlled	353	No significant differences compared to placebo group	[151]
	Calcium gluconate, 1 g; magnesium sulfate, 1 g (pre- and post-oxaliplatin)	Oxaliplatin	Randomized, double-blind, placebo-controlled, cross-over	19	No significant differences compared to placebo group	[152]
	*N/A*	Oxaliplatin	Meta-analysis	694	No significant differences compared to control group RRs (95% CI) of the incidence of ≥ Grade 2 neuropathy and ≥ Grade 1 chronic neuropathy were 0.81 (0.60–1.11) and 0.95 (0.69–1.32), respectively.	[153]
Goshajinkigan	7.5 g/day	Oxaliplatin	Randomized, controlled	45	Significant improvement in incidence of ≥ Grade 2 neuropathy compared control group	[154]
	7.5 g/day	Oxaliplatin	Randomized, double-blind, placebo-controlled	93	No significant differences compared to placebo group	[155]
	7.5 g/day	Oxaliplatin	Randomized, double-blind, placebo-controlled	188	Significant increase in incidence of ≥ Grade 2 neuropathy compared placebo group	[156]
Alpha--lipoic acid	1800 mg/day	Cisplatin or oxaliplatin	Randomized, double-blind, placebo-controlled	243	No significant differences compared to placebo group for FACT/GOG-Ntx scores, BPI scores, and patients’ functional outcomes.	[157]
Vitamin E	400 mg/day	Oxaliplatin	Randomized, controlled	65	No significant differences compared to control group	[158]
	*N/A*	Platinum, taxane or others	Meta-analysis	353	No significant differences compared to control group RR (95% CI) of incidence of neuropathy was 0.55 (0.29–1.05).	[159]
Glutathione	1500 mg/m^2^	Oxaliplatin	Randomized, double-blind, placebo-controlled	52	Significant improvements in incidence of ≥ Grade 2 neuropathy and neurophysiological findings compared placebo group	[160]
Calmangafodipir	2–10 µmol/kg	Oxaliplatin	Randomized, controlled	173	Significant improvements in Leonard scale compared to control group	[161]
Pregabalin	150–600 mg/kg	Oxaliplatin	Randomized, double-blind, placebo-controlled	199	No significant differences compared to placebo group in pain score	[162]
Minocycline	200 mg/day	Oxaliplatin	Randomized	66	No significant differences compared to control group	[163]

Abbreviations: 95% CI, 95% confidence interval; FACT/GOG-NTx, Functional Assessment of Cancer Therapy/Gynecologic Oncology Group-Neurotoxicity; RR, relative risk.

## Abbreviations

5-HT	Serotonin
95% CI	95% confidence interval
Akt	protein kinase B
ATF-3	activating transcription factor 3
ATP	adenosine triphosphate
CAT	Catalase
CaMKII	calmodulin-dependent protein kinase II
DRG	dorsal root ganglia
ERK	extracellular signal-regulated kinase
ETA	endothelin A
ETB	endothelin B
FACT/GOG-NTx	Functional Assessment of Cancer Therapy/Gynecologic Oncology Group-Neurotoxicity
GENIUS	goshajinkigan effect for oxaliplatin neurotoxicity inhibition using mFOLFOX6 regimen
GFAP	glial fibrillary acidic protein
GLT-1	glutamate transporter 1
GONE	goshajinkigan oxaliplatin neurotoxicity evaluation
GPx	glutathione peroxidase
HCN1	hyperpolarization-activated, cyclic nucleotide-gated cation channel 1
HCN2	hyperpolarization-activated, cyclic nucleotide-gated cation channel 2
HIF-1	hypoxia inducible factor 1
HMGB1	high mobility group box 1
Iba-1	ionized calcium binding adaptor protein 1
i.c.v.	intracerebroventriculary
IENF	intra-epidermal nerve fibers
IL-1β	interleukin-1 beta
IL-6	interleukin-6
i.m.	intramuscular
i.t.	intrathecal
JSPS	Japan Society for the Promotion of Science
MAPK14	mitogen-activated protein kinase-14
MBP	myelin basic protein
MEK1/2	mitogen-activated protein kinase kinases 1 and 2
MMP9/2	matrix metalloproteinase-9 and -2
mTOR	mammalian target of rapamycin
nAChR	nicotinic acetylcholine receptor
NF-κB	nuclear factor kappa-B
NMDA	N-methyl-D-aspartate
OCT2	organic cation transporter 2
OCTN1	organic cation transporter novel type 1
PDE	phosphodiesterase
PI3K	phosphatidylinositol-3 kinase
PKC	protein kinase C
RR	relative risk
SOD	superoxide dismutase
S1P	sphingosine-1-phosphate
TAFI	thrombin-activatable fibrinolysis inhibitor
TNF-α	tumor necrosis factor-α
TREK-1	tandem pore domains in weak rectifying K^+^ channel (TWIK)-related K^+^ channel 1
TRPA1	transient receptor potential ankyrin 1
TRPM8	transient receptor potential melastatin 8
TRPV1	transient receptor potential vanilloid 1
VEGF	vascular endothelial growth factor

## References

[1] Wilson R.H., Lehky T., Thomas R.R., Quinn M.G., Floeter M.K., Grem J.L. (2002). Acute oxaliplatin-induced peripheral nerve hyperexcitability. J. Clin. Oncol..

[2] Argyriou A.A., Bruna J., Marmiroli P., Cavaletti G. (2012). Chemotherapy-induced peripheral neurotoxicity (CIPN): An update. Crit. Rev. Oncol. Hematol..

[3] Pasetto L.M., D’Andrea M.R., Rossi E., Monfardini S. (2006). Oxaliplatin-related neurotoxicity: How and why?. Crit. Rev Oncol. Hematol..

[4] Sittl R., Lampert A., Huth T., Schuy E.T., Link A.S., Fleckenstein J., Alzheimer C., Grafe P., Carr R.W. (2012). Anticancer drug oxaliplatin induces acute cooling-aggravated neuropathy via sodium channel subtype Na(V)1.6-resurgent and persistent current. Proc. Natl. Acad. Sci. USA.

[5] Kagiava A., Tsingotjidou A., Emmanouilides C., Theophilidis G. (2008). The effects of oxaliplatin, an anticancer drug, on potassium channels of the peripheral myelinated nerve fibres of the adult rat. Neurotoxicology.

[6] Nakagawa T., Kaneko S. (2017). Roles of Transient Receptor Potential Ankyrin 1 in Oxaliplatin-Induced Peripheral Neuropathy. Biol. Pharm. Bull..

[7] Lehky T.J., Leonard G.D., Wilson R.H., Grem J.L., Floeter M.K. (2004). Oxaliplatin-induced neurotoxicity: Acute hyperexcitability and chronic neuropathy. Muscle Nerve..

[8] Jamieson S.M., Liu J., Connor B., McKeage M.J. (2005). Oxaliplatin causes selective atrophy of a subpopulation of dorsal root ganglion neurons without inducing cell loss. Cancer Chemother. Pharmacol..

[9] Tsutsumi K., Yamashita Y., Ushio S., Kawashiri T., Kaname T., Fujita S., Oishi R., Egashira N. (2014). Oxaliplatin induces hypomyelination and reduced neuregulin 1 expression in the rat sciatic nerve. Neurosci. Res..

[10] Loprinzi C.L., Lacchetti C., Bleeker J., Cavaletti G., Chauhan C., Hertz D.L., Kelley M.R., Lavino A., Lustberg M.B., Paice J.A. (2020). Prevention and Management of Chemotherapy-Induced Peripheral Neuropathy in Survivors of Adult Cancers: ASCO Guideline Update. J. Clin. Oncol..

[11] Ling B., Authier N., Balayssac D., Eschalier A., Coudore F. (2007). Behavioral and pharmacological description of oxaliplatin-induced painful neuropathy in rat. Pain.

[12] Ta L.E., Low P.A., Windebank A.J. (2009). Mice with cisplatin and oxaliplatin-induced painful neuropathy develop distinct early responses to thermal stimuli. Mol. Pain.

[13] Sakurai M., Egashira N., Kawashiri T., Yano T., Ikesue H., Oishi R. (2009). Oxaliplatin-induced neuropathy in the rat: Involvement of oxalate in cold hyperalgesia but not mechanical allodynia. Pain.

[14] Ghirardi O., Lo Giudice P., Pisano C., Vertechy M., Bellucci A., Vesci L., Cundari S., Miloso M., Rigamonti L.M., Nicolini G. (2005). Acetyl-L-Carnitine prevents and reverts experimental chronic neurotoxicity induced by oxaliplatin, without altering its antitumor properties. Anticancer. Res..

[15] Joseph E.K., Chen X., Bogen O., Levine J.D. (2008). Oxaliplatin acts on IB4-positive nociceptors to induce an oxidative stress-dependent acute painful peripheral neuropathy. J. Pain..

[16] Zheng H., Xiao W.H., Bennett G.J. (2011). Functional deficits in peripheral nerve mitochondria in rats with paclitaxel- and oxaliplatin-evoked painful peripheral neuropathy. Exp. Neurol..

[17] Canta A., Chiorazzi A., Pozzi E., Fumagalli G., Monza L., Meregalli C., Cavaletti G. (2020). Calmangafodipir reduces sensory alterations and prevents intraepidermal nerve fibers loss in a mouse model of oxaliplatin induced peripheral neurotoxicity. Antioxidants.

[18] Areti A., Komirishetty P., Kumar A. (2017). Carvedilol prevents functional deficits in peripheral nerve mitochondria of rats with oxaliplatin-evoked painful peripheral neuropathy. Toxicol. Appl. Pharmacol..

[19] Abdelhamid A.M., Mahmoud S.S., Abdelrahman A.E., Said N.M., Toam M., Samy W., Amer M.A. (2020). Protective effect of cerium oxide nanoparticles on cisplatin and oxaliplatin primary toxicities in male albino rats. Naunyn Schmiedebergs Arch. Pharmacol..

[20] Kawashiri T., Kobayashi D., Egashira N., Tsuchiya T., Shimazoe T. (2020). Oral administration of Cystine and Theanine ameliorates oxaliplatin-induced chronic peripheral neuropathy in rodents. Sci. Rep..

[21] Miyagi A., Kawashiri T., Shimizu S., Shigematsu N., Kobayashi D., Shimazoe T. (2019). Dimethyl Fumarate Attenuates Oxaliplatin-Induced Peripheral Neuropathy without Affecting the Anti-tumor Activity of Oxaliplatin in Rodents. Biol. Pharm Bull..

[22] Kawashiri T., Shimizu S., Shigematsu N., Kobayashi D., Shimazoe T. (2019). Donepezil ameliorates oxaliplatin-induced peripheral neuropathy via a neuroprotective effect. J. Pharmacol. Sci..

[23] Lee M., Cho S., Roh K., Chae J., Park J.H., Park J., Lee M.A., Kim J., Auh C.K., Yeom C.H. (2017). Glutathione alleviated peripheral neuropathy in oxaliplatin-treated mice by removing aluminum from dorsal root ganglia. Am. J. Transl. Res..

[24] Celik H., Kucukler S., Ozdemir S., Comakli S., Gur C., Kandemir F.M., Yardim A. (2020). Lycopene protects against central and peripheral neuropathy by inhibiting oxaliplatin-induced ATF-6 pathway, apoptosis, inflammation and oxidative stress in brains and sciatic tissues of rats. Neurotoxicology.

[25] Waseem M., Tabassum H., Parvez S. (2016). Neuroprotective effects of melatonin as evidenced by abrogation of oxaliplatin induced behavioral alterations, mitochondrial dysfunction and neurotoxicity in rat brain. Mitochondrion.

[26] Janes K., Doyle T., Bryant L., Esposito E., Cuzzocrea S., Ryerse J., Bennett G.J., Salvemini D. (2013). Bioenergetic deficits in peripheral nerve sensory axons during chemotherapy-induced neuropathic pain resulting from peroxynitrite-mediated post-translational nitration of mitochondrial superoxide dismutase. Pain.

[27] Di Cesare Mannelli L., Zanardelli M., Landini I., Pacini A., Ghelardini C., Mini E., Bencini A., Valtancoli B., Failli P. (2016). Effect of the SOD mimetic MnL4 on in vitro and in vivo oxaliplatin toxicity: Possible aid in chemotherapy induced neuropathy. Free Radic. Biol. Med..

[28] Cerles O., Benoit E., Chéreau C., Chouzenoux S., Morin F., Guillaumot M.A., Coriat R., Kavian N., Loussier T., Santulli P. (2017). Niclosamide Inhibits Oxaliplatin Neurotoxicity while Improving Colorectal Cancer Therapeutic Response. Mol. Cancer Ther..

[29] Kim S.T., Chung Y.H., Lee H.S., Chung S.J., Lee J.H., Sohn U.D., Shin Y.K., Park E.S., Kim H.C., Bang J.S. (2015). Protective effects of phosphatidylcholine on oxaliplatin-induced neuropathy in rats. Life Sci..

[30] Schwingel T.E., Klein C.P., Nicoletti N.F., Dora C.L., Hadrich G., Bica C.G., Lopes T.G., da Silva V.D., Morrone F.B. (2014). Effects of the compounds resveratrol, rutin, quercetin, and quercetin nanoemulsion on oxaliplatin-induced hepatotoxicity and neurotoxicity in mice. Naunyn Schmiedebergs Arch. Pharmacol..

[31] Azevedo M.I., Pereira A.F., Nogueira R.B., Rolim F.E., Brito G.A., Wong D.V., Lima-Júnior R.C., de Albuquerque Ribeiro R., Vale M.L. (2013). The antioxidant effects of the flavonoids rutin and quercetin inhibit oxaliplatin-induced chronic painful peripheral neuropathy. Mol. Pain.

[32] Zanardelli M., Micheli L., Cinci L., Failli P., Ghelardini C., Di Cesare Mannelli L. (2014). Oxaliplatin neurotoxicity involves peroxisome alterations. PPARγ agonism as preventive pharmacological approach. PLoS ONE.

[33] Areti A., Komirishetty P., Kalvala A.K., Nellaiappan K., Kumar A. (2018). Rosmarinic Acid Mitigates Mitochondrial Dysfunction and Spinal Glial Activation in Oxaliplatin-induced Peripheral Neuropathy. Mol. Neurobiol..

[34] Di Cesare Mannelli L., Zanardelli M., Failli P., Ghelardini C. (2012). Oxaliplatin-induced neuropathy: Oxidative stress as pathological mechanism. Prot. eff. silibinin. J. Pain..

[35] Toyama S., Shimoyama N., Szeto H.H., Schiller P.W., Shimoyama M. (2018). Protective Effect of a Mitochondria-Targeted Peptide against the Development of Chemotherapy-Induced Peripheral Neuropathy in Mice. ACS Chem. Neurosci..

[36] Toyama S., Shimoyama N., Ishida Y., Koyasu T., Szeto H.H., Shimoyama M. (2014). Characterization of acute and chronic neuropathies induced by oxaliplatin in mice and differential effects of a novel mitochondria-targeted antioxidant on the neuropathies. Anesthesiology.

[37] Yang Y., Luo L., Cai X., Fang Y., Wang J., Chen G., Yang J., Zhou Q., Sun X., Cheng X. (2018). Nrf2 inhibits oxaliplatin-induced peripheral neuropathy via protection of mitochondrial function. Free Radic. Biol. Med..

[38] Micheli L., Mattoli L., Maidecchi A., Pacini A., Ghelardini C., Di Cesare Mannelli L. (2018). Effect of Vitis vinifera hydroalcoholic extract against oxaliplatin neurotoxicity: In vitro and in vivo evidence. Sci. Rep..

[39] Li D., Kim W., Shin D., Jung Y., Bae H., Kim S.K. (2016). Preventive Effects of Bee Venom Derived Phospholipase A_2_ on Oxaliplatin-Induced Neuropathic Pain in Mice. Toxins.

[40] Di Cesare Mannelli L., Pacini A., Micheli L., Tani A., Zanardelli M., Ghelardini C. (2014). Glial role in oxaliplatin-induced neuropathic pain. Exp. Neurol..

[41] Cheng X., Huo J., Wang D., Cai X., Sun X., Lu W., Yang Y., Hu C., Wang X., Cao P. (2017). Herbal Medicine AC591 Prevents Oxaliplatin-Induced Peripheral Neuropathy in Animal Model and Cancer Patients. Front. Pharmacol..

[42] Wan C.F., Zheng L.L., Liu Y., Yu X. (2016). Houttuynia cordata Thunb reverses oxaliplatin-induced neuropathic pain in rat by regulating Th17/Treg balance. Am. J. Transl. Res..

[43] Robinson C.R., Zhang H., Dougherty P.M. (2014). Astrocytes, but not microglia, are activated in oxaliplatin and bortezomib-induced peripheral neuropathy in the rat. Neuroscience.

[44] Duan Z., Su Z., Wang H., Pang X. (2018). Involvement of pro-inflammation signal pathway in inhibitory effects of rapamycin on oxaliplatin-induced neuropathic pain. Mol. Pain.

[45] Egashira N., Hirakawa S., Kawashiri T., Yano T., Ikesue H., Oishi R. (2010). Mexiletine reverses oxaliplatin-induced neuropathic pain in rats. J. Pharmacol. Sci..

[46] Zhao M., Nakamura S., Miyake T., So K., Shirakawa H., Tokuyama S., Narita M., Nakagawa T., Kaneko S. (2014). Pharmacological characterization of standard analgesics on oxaliplatin-induced acute cold hypersensitivity in mice. J. Pharmacol. Sci..

[47] Rapacz A., Obniska J., Koczurkiewicz P., Wójcik-Pszczoła K., Siwek A., Gryboś A., Rybka S., Karcz A., Pękala E., Filipek B. (2018). Antiallodynic and antihyperalgesic activity of new 3,3-diphenyl-propionamides with anticonvulsant activity in models of pain in mice. Eur. J. Pharmacol..

[48] Deuis J.R., Lim Y.L., Rodrigues de Sousa S., Lewis R.J., Alewood P.F., Cabot P.J., Vetter I. (2014). Analgesic effects of clinically used compounds in novel mouse models of polyneuropathy induced by oxaliplatin and cisplatin. Neuro Oncol..

[49] Furgała-Wojas A., Kowalska M., Nowaczyk A., Fijałkowski Ł., Sałat K. (2020). Comparison of Bromhexine and its Active Metabolite—Ambroxol as Potential Analgesics Reducing Oxaliplatin-Induced Neuropathic Pain—Pharmacodynamic and Molecular Docking Studies. Curr. Drug Metab..

[50] Lucarini E., Micheli L., Trallori E., Citi V., Martelli A., Testai L., De Nicola G.R., Iori R., Calderone V., Ghelardini C. (2018). Effect of glucoraphanin and sulforaphane against chemotherapy-induced neuropathic pain: Kv7 potassium channels modulation by H2 S release in vivo. Phytother. Res..

[51] Di Cesare Mannelli L., Lucarini E., Micheli L., Mosca I., Ambrosino P., Soldovieri M.V., Martelli A., Testai L., Taglialatela M., Calderone V. (2017). Effects of natural and synthetic isothiocyanate-based H2S-releasers against chemotherapy-induced neuropathic pain: Role of Kv7 potassium channels. Neuropharmacology.

[52] Poupon L., Lamoine S., Pereira V., Barriere D.A., Lolignier S., Giraudet F., Aissouni Y., Meleine M., Prival L., Richard D. (2018). Targeting the TREK-1 potassium channel via riluzole to eliminate the neuropathic and depressive-like effects of oxaliplatin. Neuropharmacology.

[53] Ohsawa M., Otake S., Murakami T., Yamamoto S., Makino T., Ono H. (2014). Gabapentin prevents oxaliplatin-induced mechanical hyperalgesia in mice. J. Pharmacol. Sci..

[54] Aoki M., Kurauchi Y., Mori A., Nakahara T., Sakamoto K., Ishii K. (2014). Comparison of the effects of single doses of elcatonin and pregabalin on oxaliplatin-induced cold and mechanical allodynia in rats. Biol. Pharm Bull..

[55] Potenzieri A., Riva B., Rigolio R., Chiorazzi A., Pozzi E., Ballarini E., Cavaletti G., Genazzani A.A. (2020). Oxaliplatin-induced neuropathy occurs through impairment of haemoglobin proton buffering and is reversed by carbonic anhydrase inhibitors. Pain.

[56] Andoh T., Mizoguchi S., Kuraishi Y. (2016). Shakuyakukanzoto attenuates oxaliplatin-induced cold dysesthesia by inhibiting the expression of transient receptor potential melastatin 8 in mice. J. Tradit. Complement. Med..

[57] Mizuno K., Kono T., Suzuki Y., Miyagi C., Omiya Y., Miyano K., Kase Y., Uezono Y. (2014). Goshajinkigan, a traditional Japanese medicine, prevents oxaliplatin-induced acute peripheral neuropathy by suppressing functional alteration of TRP channels in rat. J. Pharmacol. Sci..

[58] Kato Y., Tateai Y., Ohkubo M., Saito Y., Amagai S.Y., Kimura Y.S., Iimura N., Okada M., Matsumoto A., Mano Y. (2014). Gosha-jinki-gan reduced oxaliplatin-induced hypersensitivity to cold sensation and its effect would be related to suppression of the expression of TRPM8 and TRPA1 in rats. Anticancer Drugs..

[59] Aoki M., Mori A., Nakahara T., Sakamoto K., Ishii K. (2012). Effect of synthetic eel calcitonin, elcatonin, on cold and mechanical allodynia induced by oxaliplatin and paclitaxel in rats. Eur. J. Pharmacol..

[60] Kawashiri T., Egashira N., Kurobe K., Tsutsumi K., Yamashita Y., Ushio S., Yano T., Oishi R. (2012). L type Ca²^+^ channel blockers prevent oxaliplatin-induced cold hyperalgesia and TRPM8 overexpression in rats. Mol. Pain.

[61] Resta F., Micheli L., Laurino A., Spinelli V., Mello T., Sartiani L., Di Cesare Mannelli L., Cerbai E., Ghelardini C., Romanelli M.N. (2018). Selective HCN1 block as a strategy to control oxaliplatin-induced neuropathy. Neuropharmacology.

[62] Dini L., Del Lungo M., Resta F., Melchiorre M., Spinelli V., Di Cesare Mannelli L., Ghelardini C., Laurino A., Sartiani L., Coppini R. (2018). Selective Blockade of HCN1/HCN2 Channels as a Potential Pharmacological Strategy against Pain. Front. Pharmacol..

[63] Micheli L., Di Cesare Mannelli L., Del Bello F., Giannella M., Piergentili A., Quaglia W., Carrino D., Pacini A., Ghelardini C. (2020). The Use of the Selective Imidazoline I1 Receptor Agonist Carbophenyline as a Strategy for Neuropathic Pain Relief: Preclinical Evaluation in a Mouse Model of Oxaliplatin-Induced Neurotoxicity. Neurotherapeutics.

[64] Yamamoto S., Ushio S., Egashira N., Kawashiri T., Mitsuyasu S., Higuchi H., Ozawa N., Masuguchi K., Ono Y., Masuda S. (2017). Excessive spinal glutamate transmission is involved in oxaliplatin-induced mechanical allodynia: A possibility for riluzole as a prophylactic drug. Sci. Rep..

[65] Fariello R.G., Ghelardini C., Di Cesare Mannelli L., Bonanno G., Pittaluga A., Milanese M., Misiano P., Farina C. (2014). Broad spectrum and prolonged efficacy of dimiracetam in models of neuropathic pain. Neuropharmacology.

[66] Wozniak K.M., Wu Y., Vornov J.J., Lapidus R., Rais R., Rojas C., Tsukamoto T., Slusher B.S. (2012). The orally active glutamate carboxypeptidase II inhibitor E2072 exhibits sustained nerve exposure and attenuates peripheral neuropathy. J. Pharmacol. Exp. Ther..

[67] Zhou H.H., Zhang L., Zhang H.X., Xu B.R., Zhang J.P., Zhou Y.J., Qian X.P., Ge W.H. (2019). Tat-HA-NR2B9c attenuate oxaliplatin-induced neuropathic pain. Exp. Neurol..

[68] Liu X., Zhang G., Dong L., Wang X., Sun H., Shen J., Li W., Xu J. (2013). Repeated administration of mirtazapine attenuates oxaliplatin-induced mechanical allodynia and spinal NR2B up-regulation in rats. Neurochem. Res..

[69] Mihara Y., Egashira N., Sada H., Kawashiri T., Ushio S., Yano T., Ikesue H., Oishi R. (2011). Involvement of spinal NR2B-containing NMDA receptors in oxaliplatin-induced mechanical allodynia in rats. Mol. Pain.

[70] Sada H., Egashira N., Ushio S., Kawashiri T., Shirahama M., Oishi R. (2012). Repeated administration of amitriptyline reduces oxaliplatin-induced mechanical allodynia in rats. J. Pharmacol. Sci..

[71] Shirahama M., Ushio S., Egashira N., Yamamoto S., Sada H., Masuguchi K., Kawashiri T., Oishi R. (2012). Inhibition of Ca2+/calmodulin-dependent protein kinase II reverses oxaliplatin-induced mechanical allodynia in rats. Mol. Pain.

[72] Ogihara T., Nakagawa T., Hayashi M., Koyanagi M., Yonezawa A., Omura T., Nakagawa S., Kitada N., Imai S., Matsubara K. (2019). Improvement of peripheral vascular impairment by a phosphodiesterase type 5 inhibitor tadalafil prevents oxaliplatin-induced peripheral neuropathy in mice. J. Pharmacol. Sci..

[73] Johnston I.N., Tan M., Cao J., Matsos A., Forrest D.R.L., Si E., Fardell J.E., Hutchinson M.R. (2017). Ibudilast reduces oxaliplatin-induced tactile allodynia and cognitive impairments in rats. Behav. Brain Res..

[74] Pontes R.B., Lisboa M.R.P., Pereira A.F., Lino J.A., de Oliveira F.F.B., de Mesquita A.K.V., de Freitas Alves B.W., Lima-Júnior R.C.P., Vale M.L. (2019). Involvement of Endothelin Receptors in Peripheral Sensory Neuropathy Induced by Oxaliplatin in Mice. Neurotox. Res..

[75] King K.M., Myers A.M., Soroka-Monzo A.J., Tuma R.F., Tallarida R.J., Walker E.A., Ward S.J. (2017). Single and combined effects of Δ9 -tetrahydrocannabinol and cannabidiol in a mouse model of chemotherapy-induced neuropathic pain. Br. J. Pharmacol..

[76] Gris G., Portillo-Salido E., Aubel B., Darbaky Y., Deseure K., Vela J.M., Merlos M., Zamanillo D. (2016). The selective sigma-1 receptor antagonist E-52862,enuates neuropathic pain of different aetiology in rats. Sci. Rep..

[77] Tomohisa M., Junpei O., Aki M., Masato H., Mika F., Kazumi Y., Teruo H., Tsutomu S. (2015). Possible involvement of the Sigma-1 receptor chaperone in chemotherapeutic-induced neuropathic pain. Synapse.

[78] Kanbara T., Nakamura A., Takasu K., Ogawa K., Shibasaki M., Mori T., Suzuki T., Hasegawa M., Sakaguchi G., Kanemasa T. (2014). The contribution of Gi/o protein to opioid antinociception in an oxaliplatin-induced neuropathy rat model. J. Pharmacol. Sci..

[79] Bedini A., Di Cesare Mannelli L., Micheli L., Baiula M., Vaca G., De Marco R., Gentilucci L., Ghelardini C., Spampinato S. (2020). Functional Selectivity and Antinociceptive Effects of a Novel KOPr Agonist. Front. Pharmacol..

[80] Shidahara Y., Ogawa S., Nakamura M., Nemoto S., Awaga Y., Takashima M., Hama A., Matsuda A., Takamatsu H. (2016). Pharmacological comparison of a nonhuman primate and a rat model of oxaliplatin-induced neuropathic cold hypersensitivity. Pharmacol. Res. Perspect..

[81] Furgała A., Sałat R., Sałat K. (2018). Acute cold allodynia induced by oxaliplatin is attenuated by amitriptyline. Acta Neurobiol. Exp. (Wars).

[82] Yeo J.H., Yoon S.Y., Kwon S.K., Kim S.J., Lee J.H., Beitz A.J., Roh D.H. (2016). Repetitive Acupuncture Point Treatment with Diluted Bee Venom Relieves Mechanical Allodynia and Restores Intraepidermal Nerve Fiber Loss in Oxaliplatin-Induced Neuropathic Mice. J. Pain.

[83] Kim W., Kim M.J., Go D., Min B.I., Na H.S., Kim S.K. (2016). Combined Effects of Bee Venom Acupuncture and Morphine on Oxaliplatin-Induced Neuropathic Pain in Mice. Toxins.

[84] Lim B.S., Moon H.J., Li D.X., Gil M., Min J.K., Lee G., Bae H., Kim S.K., Min B.I. (2013). Effect of bee venom acupuncture on oxaliplatin-induced cold allodynia in rats. Evid. Based Complement. Alternat. Med..

[85] Li D., Lee Y., Kim W., Lee K., Bae H., Kim S.K. (2015). Analgesic Effects of Bee Venom Derived Phospholipase A(2) in a Mouse Model of Oxaliplatin-Induced Neuropathic Pain. Toxins.

[86] Yeo J.H., Yoon S.Y., Kim S.J., Oh S.B., Lee J.H., Beitz A.J., Roh D.H. (2016). Clonidine, an alpha-2 adrenoceptor agonist relieves mechanical allodynia in oxaliplatin-induced neuropathic mice; potentiation by spinal p38 MAPK inhibition without motor dysfunction and hypotension. Int. J. Cancer..

[87] Kim W., Chung Y., Choi S., Min B.I., Kim S.K. (2017). Duloxetine Protects against Oxaliplatin-Induced Neuropathic Pain and Spinal Neuron Hyperexcitability in Rodents. Int. J. Mol. Sci..

[88] Balayssac D., Ling B., Ferrier J., Pereira B., Eschalier A., Authier N. (2014). Assessment of thermal sensitivity in rats using the thermal place preference test: Description and application in the study of oxaliplatin-induced acute thermal hypersensitivity and inflammatory pain models. Behav. Pharmacol..

[89] Baptista-de-Souza D., Di Cesare Mannelli L., Zanardelli M., Micheli L., Nunes-de-Souza R.L., Canto-de-Souza A., Ghelardini C. (2014). Serotonergic modulation in neuropathy induced by oxaliplatin: Effect on the 5HT2C receptor. Eur. J. Pharmacol..

[90] Choi S., Chae H.K., Heo H., Hahm D.H., Kim W., Kim S.K. (2019). Analgesic Effect of Melittin on Oxaliplatin-Induced Peripheral Neuropathy in Rats. Toxins.

[91] Sałat K., Kołaczkowski M., Furgała A., Rojek A., Śniecikowska J., Varney M.A., Newman-Tancredi A. (2017). Antinociceptive, antiallodynic and antihyperalgesic effects of the 5-HT1A receptor selective agonist, NLX-112 in mouse models of pain. Neuropharmacology.

[92] Yoon S.Y., Lee J.Y., Roh D.H., Oh S.B. (2018). Pharmacopuncture With Scolopendra subspinipes Suppresses Mechanical Allodynia in Oxaliplatin-Induced Neuropathic Mice and Potentiates Clonidine-induced Anti-allodynia Without Hypotension or Motor Impairment. J. Pain.

[93] Andoh T., Sakamoto A., Kuraishi Y. (2016). 5-HT1A receptor agonists, xaliproden and tandospirone, inhibit the increase in the number of cutaneous mast cells involved in the exacerbation of mechanical allodynia in oxaliplatin-treated mice. J. Pharmacol. Sci..

[94] Micov A.M., Tomić M.A., Todorović M.B., Vuković M.J., Pecikoza U.B., Jasnic N.I., Djordjevic J.D., Stepanović-Petrović R.M. (2020). Vortioxetine reduces pain hypersensitivity and associated depression-like behavior in mice with oxaliplatin-induced neuropathy. Prog. Neuropsychopharmacol. Biol. Psychiatry.

[95] Kanat O., Bagdas D., Ozboluk H.Y., Gurun M.S. (2013). Preclinical evidence for the antihyperalgesic activity of CDP-choline in oxaliplatin-induced neuropathic pain. J. BUON.

[96] Di Cesare Mannelli L., Pacini A., Matera C., Zanardelli M., Mello T., De Amici M., Dallanoce C., Ghelardini C. (2014). Involvement of α7 nAChR subtype in rat oxaliplatin-induced neuropathy: Effects of selective activation. Neuropharmacology.

[97] Wang H., Li X., Zhangsun D., Yu G., Su R., Luo S. (2019). The α9α10 Nicotinic Acetylcholine Receptor Antagonist αO-Conotoxin GeXIVA[1,2] Alleviates and Reverses Chemotherapy-Induced Neuropathic Pain. Mar. Drugs.

[98] Pacini A., Micheli L., Maresca M., Branca J.J., McIntosh J.M., Ghelardini C., Di Cesare Mannelli L. (2016). The α9α10 nicotinic receptor antagonist α-conotoxin RgIA prevents neuropathic pain induced by oxaliplatin treatment. Exp. Neurol..

[99] Huang K.M., Leblanc A.F., Uddin M.E., Kim J.Y., Chen M., Eisenmann E.D., Gibson A.A., Li Y., Hong K.W., DiGiacomo D. (2020). Neuronal uptake transporters contribute to oxaliplatin neurotoxicity in mice. J. Clin. Investig..

[100] Nishida K., Takeuchi K., Hosoda A., Sugano S., Morisaki E., Ohishi A., Nagasawa K. (2018). Ergothioneine ameliorates oxaliplatin-induced peripheral neuropathy in rats. Life Sci..

[101] Toyama S., Shimoyama N., Shimoyama M. (2017). The analgesic effect of orexin-A in a murine model of chemotherapy-induced neuropathic pain. Neuropeptides.

[102] Chaumette T., Chapuy E., Berrocoso E., Llorca-Torralba M., Bravo L., Mico J.A., Chalus M., Eschalier A., Ardid D., Marchand F. (2018). Effects of S 38093, an antagonist/inverse agonist of histamine H3 receptors, in models of neuropathic pain in rats. Eur. J. Pain..

[103] Tsubaki M., Takeda T., Matsumoto M., Kato N., Asano R.T., Imano M., Satou T., Nishida S. (2018). Trametinib suppresses chemotherapy-induced cold and mechanical allodynia via inhibition of extracellular-regulated protein kinase 1/2 activation. Am. J. Cancer Res..

[104] Tsubaki M., Takeda T., Tani T., Shimaoka H., Suzuyama N., Sakamoto K., Fujita A., Ogawa N., Itoh T., Imano M. (2015). PKC/MEK inhibitors suppress oxaliplatin-induced neuropathy and potentiate the antitumor effects. Int. J. Cancer.

[105] Janes K., Little J.W., Li C., Bryant L., Chen C., Chen Z., Kamocki K., Doyle T., Snider A., Esposito E. (2014). The development and maintenance of paclitaxel-induced neuropathic pain require activation of the sphingosine 1-phosphate receptor subtype 1. J. Biol. Chem..

[106] Minami T., Takeda M., Sata M., Kato H., Yano K., Sakai T., Tsujita R., Kawasaki K., Ito A. (2020). Thrombomodulin alfa prevents oxaliplatin-induced neuropathic symptoms through activation of thrombin-activatable fibrinolysis inhibitor and protein C without affecting anti-tumor activity. Eur. J. Pharmacol..

[107] Tsubota M., Fukuda R., Hayashi Y., Miyazaki T., Ueda S., Yamashita R., Koike N., Sekiguchi F., Wake H., Wakatsuki S. (2019). Role of non-macrophage cell-derived HMGB1 in oxaliplatin-induced peripheral neuropathy and its prevention by the thrombin/thrombomodulin system in rodents: Negative impact of anticoagulants. J. NeuroInflamm..

[108] Di Cesare Mannelli L., Tenci B., Micheli L., Vona A., Corti F., Zanardelli M., Lapucci A., Clemente A.M., Failli P., Ghelardini C. (2018). Adipose-derived stem cells decrease pain in a rat model of oxaliplatin-induced neuropathy: Role of VEGF-A modulation. Neuropharmacology.

[109] Miguel C.A., Raggio M.C., Villar M.J., Gonzalez S.L., Coronel M.F. (2019). Anti-allodynic and anti-inflammatory effects of 17α-hydroxyprogesterone caproate in oxaliplatin-induced peripheral neuropathy. J. Peripher. Nerv. Syst..

[110] Taleb O., Bouzobra F., Tekin-Pala H., Meyer L., Mensah-Nyagan A.G., Patte-Mensah C. (2017). Behavioral and electromyographic assessment of oxaliplatin-induced motor dysfunctions: Evidence for a therapeutic effect of allopregnanolone. Behav. Brain Res..

[111] Shigematsu N., Kawashiri T., Kobayashi D., Shimizu S., Mine K., Hiromoto S., Uchida M., Egashira N., Shimazoe T. (2020). Neuroprotective effect of alogliptin on oxaliplatin-induced peripheral neuropathy in vivo and in vitro. Sci. Rep..

[112] Yi J.M., Shin S., Kim N.S., Bang O.S. (2019). Neuroprotective Effects of an Aqueous Extract of Forsythia viridissima and Its Major Constituents on Oxaliplatin-Induced Peripheral Neuropathy. Molecules.

[113] Yi J.M., Shin S., Kim N.S., Bang O.S. (2019). Ameliorative effects of aqueous extract of Forsythiae suspensa fruits on oxaliplatin-induced neurotoxicity in vitro and in vivo. BMC Complement. Altern. Med..

[114] Cho E.S., Yi J.M., Park J.S., Lee Y.J., Lim C.J., Bang O.S., Kim N.S. (2016). Aqueous extract of Lithospermi radix attenuates oxaliplatin-induced neurotoxicity in both in vitro and in vivo models. BMC Complement. Altern. Med..

[115] Sałat K., Furgała A., Sałat R. (2019). Interventional and preventive effects of aripiprazole and ceftriaxone used alone or in combination on oxaliplatin-induced tactile and cold allodynia in mice. Biomed. Pharmacother..

[116] Di Cesare Mannelli L., Pacini A., Micheli L., Femia A.P., Maresca M., Zanardelli M., Vannacci A., Gallo E., Bilia A.R., Caderni G. (2017). Astragali radix: Could it be an adjuvant for oxaliplatin-induced neuropathy?. Sci. Rep..

[117] Cerles O., Gonçalves T.C., Chouzenoux S., Benoit E., Schmitt A., Bennett Saidu N.E., Kavian N., Chéreau C., Gobeaux C., Weill B. (2019). Preventive action of benztropine on platinum-induced peripheral neuropathies and tumor growth. Acta Neuropathol. Commun..

[118] Kim C., Lee J.H., Kim W., Li D., Kim Y., Lee K., Kim S.K. (2016). The Suppressive Effects of Cinnamomi Cortex and Its Phytocompound Coumarin on Oxaliplatin-Induced Neuropathic Cold Allodynia in Rats. Molecules.

[119] Di Cesare Mannelli L., Piccolo M., Maione F., Ferraro M.G., Irace C., De Feo V., Ghelardini C., Mascolo N. (2018). Tanshinones from Salvia miltiorrhiza Bunge revert chemotherapy-induced neuropathic pain and reduce glioblastoma cells malignancy. Biomed. Pharmacother..

[120] Al Moundhri M.S., Al-Salam S., Al Mahrouqee A., Beegam S., Ali B.H. (2013). The effect of curcumin on oxaliplatin and cisplatin neurotoxicity in rats: Some behavioral, biochemical, and histopathological studies. J. Med. Toxicol..

[121] Fujita S., Ushio S., Ozawa N., Masuguchi K., Kawashiri T., Oishi R., Egashira N. (2015). Exenatide Facilitates Recovery from Oxaliplatin-Induced Peripheral Neuropathy in Rats. PLoS ONE.

[122] Yamamoto S., Yamashita T., Ito M., Caaveiro J.M.M., Egashira N., Tozaki-Saitoh H., Tsuda M. (2019). New pharmacological effect of fulvestrant to prevent oxaliplatin-induced neurodegeneration and mechanical allodynia in rats. Int. J. Cancer.

[123] Mizuno K., Shibata K., Komatsu R., Omiya Y., Kase Y., Koizumi S. (2016). An effective therapeutic approach for oxaliplatin-induced peripheral neuropathy using a combination therapy with goshajinkigan and bushi. Cancer Biol. Ther..

[124] Ushio S., Egashira N., Sada H., Kawashiri T., Shirahama M., Masuguchi K., Oishi R. (2012). Goshajinkigan reduces oxaliplatin-induced peripheral neuropathy without affecting anti-tumour efficacy in rodents. Eur. J. Cancer..

[125] Yang Y., Hu L., Wang C., Yang X., Song L., Jiang C., Li Y., Li T., Liu W.T., Feng J. (2019). p38/TF/HIF-α Signaling Pathway Participates in the Progression of CIPN in Mice. Biomed Res. Int..

[126] Chiorazzi A., Wozniak K.M., Rais R., Wu Y., Gadiano A.J., Farah M.H., Liu Y., Canta A., Alberti P., Rodriguez-Menendez V. (2018). Ghrelin agonist HM01 attenuates chemotherapy-induced neurotoxicity in rodent models. Eur. J. Pharmacol..

[127] Areti A., Komirishetty P., Akuthota M., Malik R.A., Kumar A. (2017). Melatonin prevents mitochondrial dysfunction and promotes neuroprotection by inducing autophagy during oxaliplatin-evoked peripheral neuropathy. J. Pineal. Res..

[128] Martinez N.W., Sánchez A., Diaz P., Broekhuizen R., Godoy J., Mondaca S., Catenaccio A., Macanas P., Nervi B., Calvo M. (2020). Metformin protects from oxaliplatin induced peripheral neuropathy in rats. Neurobiol. Pain.

[129] Pereira A.F., Pereira L.M.S., Silva C.M.P., Freitas Alves B.W., Barbosa J.S., Pinto F.M.M., Pereira A.C., Silva K.O., Pontes R.B., Alencar N.M.N. (2019). Metformin reduces c-Fos and ATF3 expression in the dorsal root ganglia and protects against oxaliplatin-induced peripheral sensory neuropathy in mice. Neurosci. Lett..

[130] Masuguchi K., Watanabe H., Kawashiri T., Ushio S., Ozawa N., Morita H., Oishi R., Egashira N. (2014). Neurotropin^®^ relieves oxaliplatin-induced neuropathy via Gi protein-coupled receptors in the monoaminergic descending pain inhibitory system. Life Sci..

[131] Kawashiri T., Egashira N., Watanabe H., Ikegami Y., Hirakawa S., Mihara Y., Yano T., Ikesue H., Oishi R. (2011). Prevention of oxaliplatin-induced mechanical allodynia and neurodegeneration by neurotropin in the rat model. Eur. J. Pain.

[132] Suzuki T., Yamamoto A., Ohsawa M., Motoo Y., Mizukami H., Makino T. (2017). Effect of ninjin’yoeito and ginseng extracts on oxaliplatin-induced neuropathies in mice. J. Nat. Med..

[133] Di Cesare Mannelli L., Pacini A., Corti F., Boccella S., Luongo L., Esposito E., Cuzzocrea S., Maione S., Calignano A., Ghelardini C. (2015). Antineuropathic profile of N-palmitoylethanolamine in a rat model of oxaliplatin-induced neurotoxicity. PLoS ONE.

[134] Suzuki T., Miyamoto K., Yokoyama N., Sugi M., Kagioka A., Kitao Y., Adachi T., Ohsawa M., Mizukami H., Makino T. (2016). Processed aconite root and its active ingredient neoline may alleviate oxaliplatin-induced peripheral neuropathic pain. J. Ethnopharmacol..

[135] Aoki M., Mori A., Nakahara T., Sakamoto K., Ishii K. (2013). Salmon calcitonin reduces oxaliplatin-induced cold and mechanical allodynia in rats. Biol. Pharm. Bull..

[136] Cheng W., Xiang W., Wang S., Xu K. (2019). Tanshinone IIA ameliorates oxaliplatin-induced neurotoxicity via mitochondrial protection and autophagy promotion. Am. J. Transl. Res..

[137] Alberti P., Canta A., Chiorazzi A., Fumagalli G., Meregalli C., Monza L., Pozzi E., Ballarini E., Rodriguez-Menendez V., Oggioni N. (2020). Topiramate prevents oxaliplatin-related axonal hyperexcitability and oxaliplatin induced peripheral neurotoxicity. Neuropharmacology.

[138] Tenci B., Di Cesare Mannelli L., Maresca M., Micheli L., Pieraccini G., Mulinacci N., Ghelardini C. (2017). Effects of a water extract of Lepidium meyenii root in different models of persistent pain in rats. Z. Naturforsch. C J. Biosci..

[139] Deng B., Jia L., Pan L., Song A., Wang Y., Tan H., Xiang Q., Yu L., Ke D. (2016). Wen-Luo-Tong Prevents Glial Activation and Nociceptive Sensitization in a Rat Model of Oxaliplatin-Induced Neuropathic Pain. Evid. Based Complement. Alternat. Med..

[140] McQuade R.M., Carbone S.E., Stojanovska V., Rahman A., Gwynne R.M., Robinson A.M., Goodman C.A., Bornstein J.C., Nurgali K. (2016). Role of oxidative stress in oxaliplatin-induced enteric neuropathy and colonic dysmotility in mice. Br. J. Pharmacol..

[141] Di Cesare Mannelli L., Zanardelli M., Failli P., Ghelardini C. (2013). Oxaliplatin-induced oxidative stress in nervous system-derived cellular models: Could it correlate with in vivo neuropathy?. Free Radic. Biol. Med..

[142] Adelsberger H., Quasthoff S., Grosskreutz J., Lepier A., Eckel F., Lersch C. (2000). The chemotherapeutic oxaliplatin alters voltage-gated Na(+) channel kinetics on rat sensory neurons. Eur. J. Pharmacol..

[143] Grolleau F., Gamelin L., Boisdron-Celle M., Lapied B., Pelhate M., Gamelin E. (2001). A possible explanation for a neurotoxic effect of the anticancer agent oxaliplatin on neuronal voltage-gated sodium channels. J. Neurophysiol..

[144] Gauchan P., Andoh T., Kato A., Kuraishi Y. (2009). Involvement of increased expression of transient receptor potential melastatin 8 in oxaliplatin-induced cold allodynia in mice. Neurosci. Lett..

[145] Ta L.E., Bieber A.J., Carlton S.M., Loprinzi C.L., Low P.A., Windebank A.J. (2010). Transient Receptor Potential Vanilloid 1 is essential for cisplatin-induced heat hyperalgesia in mice. Mol. Pain.

[146] Descoeur J., Pereira V., Pizzoccaro A., Francois A., Ling B., Maffre V., Couette B., Busserolles J., Courteix C., Noel J. (2011). Oxaliplatin-induced cold hypersensitivity is due to remodelling of ion channel expression in nociceptors. EMBO Mol. Med..

[147] Yoshimura M., Furue H. (2006). Mechanisms for the anti-nociceptive actions of the descending noradrenergic and serotonergic systems in the spinal cord. J. Pharmacol. Sci..

[148] Smith E.M., Pang H., Cirrincione C., Fleishman S., Paskett E.D., Ahles T., Bressler L.R., Fadul C.E., Knox C., Le-Lindqwister N. (2013). Alliance for Clinical Trials in Oncology. Effect of duloxetine on pain, function, and quality of life among patients with chemotherapy-induced painful peripheral neuropathy: A randomized clinical trial. JAMA.

[149] Grothey A., Nikcevich D.A., Sloan J.A., Kugler J.W., Silberstein P.T., Dentchev T., Wender D.B., Novotny P.J., Chitaley U., Alberts S.R. (2011). Intravenous calcium and magnesium for oxaliplatin-induced sensory neurotoxicity in adjuvant colon cancer: NCCTG N04C7. J. Clin. Oncol..

[150] Grothey A., Hart L.L., Rowland K.M., Ansari R.H., Alberts S.R., Chowhan N.M., Hochster H.S. (2008). Intermittent oxaliplatin (oxali) administration and time-to-treatment-failure (TTF) in metastatic colorectal cancer (mCRC): Final results of the phase III CONcePT trial. J. Clin. Oncol..

[151] Loprinzi C.L., Qin R., Dakhil S.R., Fehrenbacher L., Flynn K.A., Atherton P., Seisler D., Qamar R., Lewis G.C., Grothey A. (2014). Phase III randomized, placebo-controlled, double-blind study of intravenous calcium and magnesium to prevent oxaliplatin-induced sensory neurotoxicity (N08CB/Alliance). J. Clin. Oncol..

[152] Han C.H., Khwaounjoo P., Kilfoyle D.H., Hill A., McKeage M.J. (2013). Phase I drug-interaction study of effects of calcium and magnesium infusions on oxaliplatin pharmacokinetics and acute neurotoxicity in colorectal cancer patients. BMC Cancer.

[153] Jordan B., Jahn F., Beckmann J., Unverzagt S., Müller-Tidow C., Jordan K. (2016). Calcium and Magnesium Infusions for the Prevention of Oxaliplatin-Induced Peripheral Neurotoxicity: A Systematic Review. Oncology.

[154] Nishioka M., Shimada M., Kurita N., Iwata T., Morimoto S., Yoshikawa K., Higashijima J., Miyatani T., Kono T. (2011). The Kampo medicine, Goshajinkigan, prevents neuropathy in patients treated by FOLFOX regimen. Int. J. Clin. Oncol..

[155] Kono T., Hata T., Morita S., Munemoto Y., Matsui T., Kojima H., Takemoto H., Fukunaga M., Nagata N., Shimada M. (2013). Goshajinkigan oxaliplatin neurotoxicity evaluation (GONE): A phase 2, multicenter, randomized, double‑blind, placebo‑controlled trial of goshajinkigan to prevent oxaliplatin‑induced neuropathy. Cancer Chemother. Pharmacol..

[156] Oki E., Emi Y., Kojima H., Higashijima J., Kato T., Miyake Y., Kon M., Ogata Y., Takahashi K., Ishida H. (2015). Preventive effect of Goshajinkigan on peripheral neurotoxicity of FOLFOX therapy (GENIUS trial): A placebo-controlled, double-blind, randomized phase III study. Int. J. Clin. Oncol..

[157] Guo Y., Jones D., Palmer J.L., Forman A., Dakhil S.R., Velasco M.R., Weiss M., Gilman P., Mills G.M., Noga S.J. (2014). Oral alpha-lipoic acid to prevent chemotherapy-induced peripheral neuropathy: A randomized, double-blind, placebo-controlled trial. Support. Care Cancer.

[158] Salehi Z., Roayaei M. (2015). Effect of Vitamin E on Oxaliplatin-induced Peripheral Neuropathy Prevention: A Randomized Controlled Trial. Int. J. Prev. Med..

[159] Huang H., He M., Liu L., Huang L. (2016). Vitamin E does not decrease the incidence of chemotherapy-induced peripheral neuropathy: A meta-analysis. Contemp. Oncol..

[160] Cascinu S., Catalano V., Cordella L., Labianca R., Giordani P., Baldelli A.M., Beretta G.D., Ubiali E., Catalano G. (2002). Neuroprotective effect of reduced glutathione on oxaliplatin-based chemotherapy in advanced colorectal cancer: A randomized, double-blind, placebo-controlled trial. J. Clin. Oncol..

[161] Glimelius B., Manojlovic N., Pfeiffer P., Mosidze B., Kurteva G., Karlberg M., Mahalingam D., Buhl Jensen P., Kowalski J., Bengtson M. (2018). Persistent prevention of oxaliplatin-induced peripheral neuropathy using calmangafodipir (PledOx^®^): A placebo-controlled randomised phase II study (PLIANT). Acta Oncol..

[162] de Andrade D.C., Jacobsen Teixeira M., Galhardoni R., Ferreira K.S.L., Braz Mileno P., Scisci N., Zandonai A., Teixeira W.G.J., Saragiotto D.F., Silva V. (2017). Pregabalin for the Prevention of Oxaliplatin-Induced Painful Neuropathy: A Randomized, Double-Blind Trial. Oncologist.

[163] Wang X.S., Shi Q., Bhadkamkar N.A., Cleeland C.S., Garcia-Gonzalez A., Aguilar J.R., Heijnen C., Eng C. (2019). Minocycline for Symptom Reduction During Oxaliplatin-Based Chemotherapy for Colorectal Cancer: A Phase II Randomized Clinical Trial. J. Pain Symptom. Manag..

